# Psychological traits as determinants of resilience: Insights from Tunisian women

**DOI:** 10.1192/j.eurpsy.2025.1614

**Published:** 2025-08-26

**Authors:** H. Mhiri, I. Chaari, I. Mannoubi, N. Boussaid, F. Charfeddine, L. Aribi, N. Messedi, J. Aloulou

**Affiliations:** 1 Psychiatric department “B”, Hedi Chaker University Hospital, Sfax, Tunisia

## Abstract

**Introduction:**

Personality encompasses stable traits, behaviors, thoughts, and emotions that shape how individuals interact with their environment. It stands to reason that personality can influence how a person responds to life’s challenges, essentially defining their level of resilience. Tunisian women, who frequently face a range of daily struggles, represent a significant group for exploring the dynamics of resilience. Understanding how their personality traits impact their ability to adapt and thrive amidst adversity provides valuable insights, making this an important area of study.

**Objectives:**

To examine the relationship between personality traits and resilience among Tunisian women.

**Methods:**

This cross-sectional study surveyed Tunisian women aged 18 and above using an online questionnaire between June and August 2024. Personality traits were assessed using the 10-item Big Five Inventory (BFI-10), while psychological resilience was measured using the 25-item Connor-Davidson Resilience Scale (CD-RISC 25).

**Results:**

Data were collected from 695 Tunisian women, with a mean age of 36.72 ± 12.23 years. Among participants, 24.7% were students, 56.5% were employed, 49.2% were married, and 50.6% had children. Regarding sexual orientation, 93.4% identified as heterosexual, 0.4% as homosexual, 3.3% as bisexual, and 2.9% as undefined. The average resilience score was 68.26 ± 14.09, with 26.3% of participants exhibiting low resilience. Mean scores for personality traits were as follows: agreeableness: 6.8 ± 1.86, extraversion: 6.1 ± 1.79, neuroticism: 6.33 ± 2.01, conscientiousness: 7.16 ± 1.94, and openness to experience: 8.03 ± 1.71. Resilience was positively associated with age (p < 10⁻³, r = 0.143), marital status (p = 0.022), sexual orientation (p = 0.001), and education level (p < 10⁻³), with 80% of those with only primary education showing low resilience compared to 24.2% of those with a university education. Personality traits showed significant correlations with resilience: agreeableness (p < 10⁻³, r = 0.165), extraversion (p < 10⁻³, r = 0.207), conscientiousness (p < 10⁻³, r = 0.367), and openness to experience (p < 10⁻³, r = 0.278) were positively correlated, while neuroticism was negatively correlated (p < 10⁻³, r = -0.482).

**Conclusions:**

Personality traits are significant determinants of resilience in Tunisian women. Positive traits like agreeableness, openness, extraversion, and conscientiousness enhance resilience, while neuroticism has the opposite effect. Considering that personality is influenced by factors such as early childhood experiences and parenting styles, future interventions could focus on fostering these positive traits to strengthen resilience.

**Disclosure of Interest:**

None Declared

